# Contribution of fat, sugar and salt to diets in the Pacific Islands: a systematic review

**DOI:** 10.1017/S1368980018003609

**Published:** 2019-01-07

**Authors:** Joseph Alvin Santos, Briar McKenzie, Kathy Trieu, Sara Farnbach, Claire Johnson, Jimaima Schultz, Anne Marie Thow, Wendy Snowdon, Colin Bell, Jacqui Webster

**Affiliations:** 1 The George Institute for Global Health, University of New South Wales, PO Box M201, Missenden Road, Sydney, NSW 2052, Australia; 2 National Drug and Alcohol Research Centre, University of New South Wales, Sydney, NSW, Australia; 3 Independent Nutrition Consultant, Suva, Fiji; 4 Menzies Centre for Health Policy, School of Public Health, The University of Sydney, Sydney, NSW, Australia; 5 Global Obesity Centre, Deakin University, Geelong, VIC, Australia

**Keywords:** Fat intake, Sugar intake, Salt intake, Pacific Island countries

## Abstract

**Objective:**

Pacific Island countries are experiencing a high burden of diet-related non-communicable diseases; and consumption of fat, sugar and salt are important modifiable risk factors contributing to this. The present study systematically reviewed and summarized available literature on dietary intakes of fat, sugar and salt in the Pacific Islands.

**Design:**

Electronic databases (PubMed, Scopus, ScienceDirect and GlobalHealth) were searched from 2005 to January 2018. Grey literature was also searched and key stakeholders were consulted for additional information. Study eligibility was assessed by two authors and quality was evaluated using a modified tool for assessing dietary intake studies.

**Results:**

Thirty-one studies were included, twenty-two contained information on fat, seventeen on sugar and fourteen on salt. Dietary assessment methods varied widely and six different outcome measures for fat, sugar and salt intake – absolute intake, household expenditure, percentage contribution to energy intake, sources, availability and dietary behaviours – were used. Absolute intake of fat ranged from 25·4 g/d in Solomon Islands to 98·9 g/d in Guam, while salt intake ranged from 5·6 g/d in Kiribati to 10·3 g/d in Fiji. Only Guam reported on absolute sugar intake (47·3 g/d). Peer-reviewed research studies used higher-quality dietary assessment methods, while reports from national surveys had better participation rates but mostly utilized indirect methods to quantify intake.

**Conclusions:**

Despite the established and growing crisis of diet-related diseases in the Pacific, there is inadequate evidence about what Pacific Islanders are eating. Pacific Island countries need nutrition monitoring systems to fully understand the changing diets of Pacific Islanders and inform effective policy interventions.

The Pacific Island region comprises a wide area of diverse nations that suffer from some of the highest rates of non-communicable diseases (NCD) globally^(^ ^1^ ^)^. NCD, including CVD and diabetes, account for about 70 % of all deaths in the region, most of which occur before the age of 60 years^(^ ^2^ ^)^. In fact, NCD are the primary cause of premature deaths in all Pacific Island countries^(^ ^1^ ^)^. Alarmingly, NCD rates are expected to increase given existing risk factors^(^ ^1^ ^)^ and the transition from traditional diets based on locally grown foods^(^ ^3^ ^,^ ^4^ ^)^ to modern diets characterized by imported foods and refined oils, sugar and confectionery, and processed meats^(^ ^5^ ^,^ ^6^ ^)^.

In 2013, the WHO recommended that all Member States develop policy measures for promoting a healthy diet, as part of efforts to reduce premature mortality from NCD by 25 % by 2025^(^ ^7^ ^)^. Recommendations include policies to reduce population intakes of salt, SFA, *trans*-fats, and free and added sugars^(^ ^7^ ^)^. In Pacific Island countries, several initiatives have already been implemented such as changes in trade policy including import bans on carbonated soft drinks in Tokelau^(^ ^8^ ^)^; sales ban on mutton flaps in Fiji^(^ ^9^ ^)^; import tariffs or excise taxes on sugary drinks in twelve Pacific Island countries^(^ ^8^ ^,^ ^10^ ^)^; regional food-based dietary guidelines^(^ ^11^ ^)^; and support for local agriculture and efforts to improve food security^(^ ^12^ ^)^. Evaluation of some of these efforts showed barriers to success including insufficient engagement from other sectors^(^ ^9^ ^)^, and there has been no evidence of significant decline in NCD rates in the region although stabilization of some risk factors is being seen^(^ ^13^ ^)^.

A good nutrition monitoring system is important for developing and strengthening policies and actions and evaluating health interventions, especially in the Pacific where resources are scarce^(^ ^14^ ^)^. As such, data on current intakes of fat, sugar and salt are needed for setting realistic goals and plans for strategies to reduce consumption of these nutrients. These data are critical to lay the foundation of evidence-based interventions and for monitoring impact of interventions. While there have been reviews on dietary patterns and intakes in Pacific Island countries^(^ ^3^ ^,^ ^15^ ^,^ ^16^ ^)^, no reviews have been done in the last 10 years. Thus, we aimed to systematically review and synthesize recent literature on dietary intakes of Pacific Island adult populations, with a specific focus on fat, sugar and salt intake.

## Methods

A search protocol was developed based on the Preferred Reporting Items for Systematic Reviews and Meta-Analyses (PRISMA) guidelines for systematic reviews. This included a defined search strategy, methods for data extraction and synthesis, and assessment of quality of the studies.

### Search protocol and databases

A search for published literature was conducted using PubMed, Scopus, Science Direct and Global Health between 2005 and January 2018, limited to English language and human studies. The search strategy comprised three groups of search terms: nutrient terms (salt, sugar and fat), intake terms (eat, consumption, ingest and intake) and country terms (each of the twenty-two Pacific Island countries and areas, Pacific Islands, and Micronesia, Polynesia or Melanesia). A grey literature search was also performed using Google Scholar, Open Grey, Prism Survey and WHO databases, limited to the first ten pages of results. Key stakeholders, including experts, researchers and relevant organizations working to address diet-related issues in the Pacific Islands, were contacted to obtain information about unpublished documents or data sets.

### Study selection

All search results were imported to EndNote X7.7.1 (Thomas Reuters, 2016) for title and abstract screening. Two authors (J.A.S. and B.M.) independently screened the titles and abstracts, and the full texts of potentially related articles were obtained and assessed further for eligibility. Disagreements at any stage of eligibility assessment (title and abstract screening, full-text review stage) were discussed by the two authors until agreement was reached. Studies not meeting the inclusion criteria as well as those not available electronically were excluded.

#### Inclusion criteria

Studies of any design were included if the primary or secondary outcomes provided information relevant to dietary patterns or preferences or intakes of fat, sugar and salt carried out on a general Pacific Island adult population (15–64 years of age). Countries of studies included were American Samoa, Commonwealth of the Northern Mariana Island (CNMI), Cook Islands, Federated States of Micronesia (FSM), Fiji, French Polynesia, Guam, Kiribati, Nauru, New Caledonia, Niue, Palau, Papua New Guinea (PNG), Pitcairn Island, Republic of the Marshall Islands (RMI), Samoa, Solomon Islands, Tokelau, Tonga, Tuvalu, Vanuatu, and Wallis and Futuna.

#### Exclusion criteria

Studies focusing on fortification, fishing, agriculture, disaster conditions, and those that included sick populations only were excluded. Studies specifically conducted in women who were lactating were excluded. Studies that only reported on data collected before 2005 were also excluded, regardless of the publication date.

### Data extraction and synthesis

Data extraction was done by two authors (J.A.S. and B.M.) using a data extraction form designed for the present review. Information on study year, country of study, research design, type of participants and sample size, dietary assessment methods, and intake (e.g. description of intake or absolute intake, if reported) of fat, sugar and salt were extracted.

Where applicable, quality of the included studies was assessed by a modified tool for evaluating dietary intake studies^(^ ^17^ ^)^. This tool was originally used to evaluate intake studies conducted among Aboriginal and Torres Strait Islanders in Australia. For the purpose of the present review, seven domains were employed, including: (i) involvement of Pacific Islanders in the study during conception, design or conduct of research; (ii) representativeness of sample of the underlying population; (iii) participation rate; (iv) reliability and validity of the dietary assessment tool; (v) quality of dietary assessment tool based on a set of criteria; (vi) accuracy of the food composition tables; and (vii) completeness and usability of results. More details on how each quality domain was scored are available in the online supplementary material, Supplemental Table 1. Two authors (J.A.S. and B.M.) assessed the quality of the studies and a third author (K.T.) was consulted for any disagreements in assessment. Each study was rated as low, high or unclear quality for each quality domain.

A narrative synthesis of findings was based on all included studies. A descriptive approach was undertaken due to the heterogeneous nature of the reported outcomes.

## Results

### Search results

**Fig. 1:**
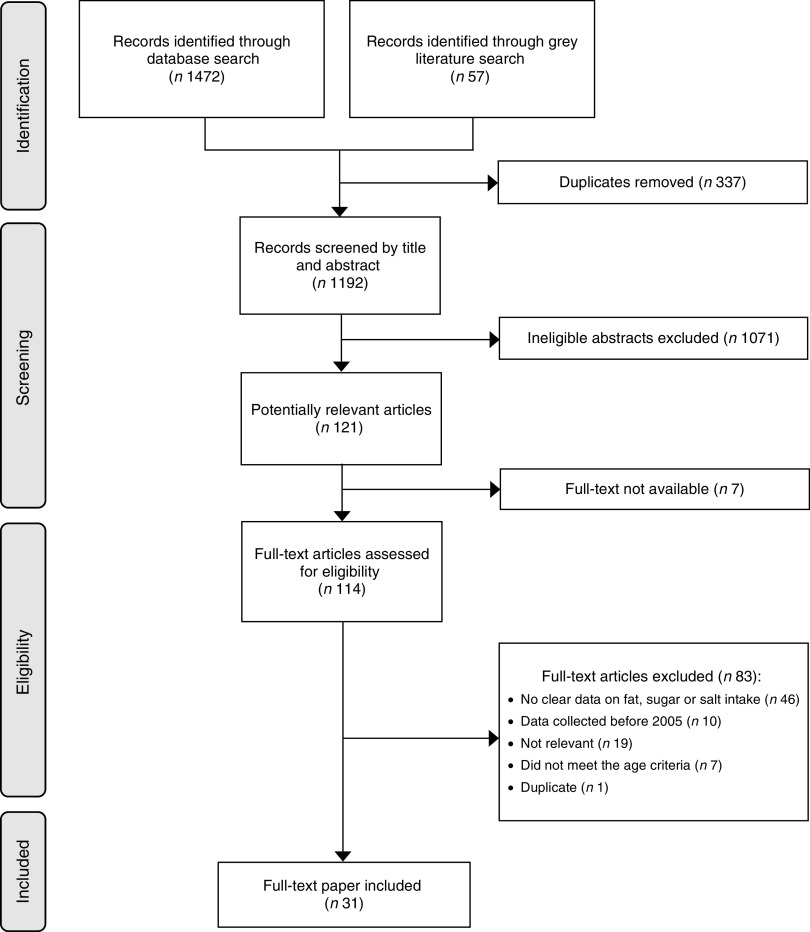
Flowchart of studies included in the present systematic review on the contribution of fat, sugar and salt to diets in the Pacific Islands

The search identified 1529 records (1472 from the peer-reviewed and fifty-seven from the grey literature search and key stakeholders), of which 121 were considered potentially relevant (Fig. 1). Of these, seven full texts were not available and eighty-three were excluded. The reasons for excluding the studies were: no clear data on fat, sugar or salt intake (*n* 46); data collected before 2005 (*n* 10); not relevant (*n* 19); did not meet the age criteria (*n* 7); and duplicate (*n* 1). Ultimately, thirty-one studies met the criteria.

### Description of the studies

Study design varied across the included studies, ranging from cross-sectional (*n* 20) to pre–post intervention studies (*n* 4), dietary modelling (*n* 4), use of secondary data (*n* 2) and case–control (*n* 1; Table 1). Three studies^(^ ^18^ ^–^ ^20^ ^)^ included women only. Eleven studies used households as the unit of analysis^(^ ^21^ ^–^ ^31^ ^)^, fourteen reported the results at the individual level^(^ ^18^ ^–^ ^20^ ^,^ ^32^ ^–^ ^40^ ^)^ (two unpublished results: Fiji National Nutrition Survey 2017 and Vanuatu Ministry of Health 2017), while the rest described intake at a population level^(^ ^41^ ^–^ ^46^ ^)^. Sample sizes ranged from 150 to 4479 households and from thirty-four to 3220 individuals. Three studies^(^ ^44^ ^–^ ^46^ ^)^ included multiple Pacific Island countries, while the others involved a single country. Among the single-country studies, six were done in Samoa; three each in FSM, Solomon Islands and Vanuatu; two each in Fiji, Guam and Tonga; and one each in CNMI, Cook Islands, Nauru, New Caledonia, Palau, PNG and Tuvalu. No data were obtained from six countries: American Samoa, French Polynesia, Niue, Pitcairn Island, Tokelau, and Wallis and Futuna. Lastly, more than half (*n* 17) of the studies were from the grey literature and these were mainly government surveys.

**Table 1:** Dietary study characteristics and reported data on fat, sugar and salt intake

				Reported data on intake		
Study, country	Description, study design	Sample characteristics	Dietary assessment method	Fat	Sugar	Salt
Dela Cruz and Cash (2016)^(^ ^32^ ^)^ CNMI	Hybrid NCD and risk factor household survey Cross-sectional	Household survey; adults aged 18 years or above; *n* 1091	NCD risk factor questionnaire		BE	BE
Statistics Office (2007)^(^ ^21^ ^)^ Cook Islands	Household Expenditure Survey Cross-sectional	773 households from three regions in Cook Islands	Two-week food diary and expenditure	EX	EX	
Statistics Division (2014)^(^ ^22^ ^)^ FSM	HIES Cross-sectional	1988 households	Two-week food diary and expenditure	EX	EX	
Hanson *et al*. (2011)^(^ ^33^ ^)^ FSM	Nutrition intervention Pre–post intervention study	One adult from each household; seventy-five households at baseline and sixty-eight at follow-up	7d Helen Keller International FFQ	BE	BE	BE
Kaufer *et al*. (2010)^(^ ^18^ ^)^ FSM	Food-based intervention Pre–post intervention study	One adult woman from each household; forty households within a Pohnpeian community	7d FFQ; two non-consecutive 24H-FR and an attitudes questionnaire	AI, PC, BE	BE	
Fiji National Nutrition Survey 2017 (unpublished results) Fiji	National Nutrition Survey Cross-sectional	3220 adults	24H-FR	AI, MS		AI, MS
Pillay *et al*. (2017)^(^ ^34^ ^)^ Fiji	Salt reduction intervention Pre–post intervention study	Adults aged 25–64 years; *n* 169 at baseline and *n* 272 at follow-up	24H-UNa			AI
Leon-Guerrero *et al*. (2015)^(^ ^35^ ^)^ Guam	Validity and reliability study Cross-sectional	Adults aged 18–61 years; *n* 109	Quantitative FFQ validated by 2d food record	AI		AI
Paulino *et al*. (2011)^(^ ^19^ ^)^ Guam	Comparative study of intake between feast and non-feast days Cross-sectional	Women aged 40 years and above living in Guam; *n* 25 Chamorro and *n* 24 Filipino	24H-FR of a previous feast day and a non-feast day	PC	AI	
Bureau of Statistics (2014)^(^ ^23^ ^)^ Nauru	HIES Cross-sectional	515 households	Two-week food diary and expenditure	EX	EX	
Baudchon (2009)^(^ ^24^ ^)^ New Caledonia	Household Consumption Survey Cross-sectional	3704 households	Record of expenses and resources of households	EX	EX	
Office of Planning and Statistics (2014)^(^ ^25^ ^)^ Palau	HIES Cross-sectional	1145 households	Two-week food diary and expenditure	EX	EX	
Temple *et al*. (2010)^(^ ^26^ ^)^ PNG	Salt iodization and consumption survey Cross-sectional	150 households in Moresby North East and Moresby South	Salt weighing			AI
Trieu *et al*. (2017)^(^ ^36^ ^)^ Samoa	Salt reduction intervention Pre–post intervention study	Adults aged 18–64 years; *n* 234 at baseline and *n* 479 at follow-up	24H-UNa			AI
Martyn *et al*. (2017)^(^ ^41^ ^)^ Samoa	Identification of household factors associated with nutrition Modelling	Data from HIES: 16 356 individuals and 2791 households	Data on household food expenditure from HIES	AV, MS	PC	AV, MS
Webster *et al*. (2016)^(^ ^37^ ^)^ Samoa	Assessment of salt intake Cross-sectional	293 individuals aged 18–64 years	24H-UNa and behaviours questionnaire			AI, BE
Samoa Bureau of Statistics (2016)^(^ ^27^ ^)^ Samoa	HIES Cross-sectional	2348 households	Two-week food diary and expenditure	EX	EX	
Land *et al*. (2016)^(^ ^20^ ^)^ Samoa	Assessment of salt intake and iodine status Cross-sectional	119 women aged 18–45 years	24H-UNa			AI
Seiden *et al*. (2012)^(^ ^42^ ^)^ Samoa	Trends in food availability and pricing Secondary analysis of data	National-level data	Data from annual food balance sheets	AV, MS		
Tsuchiya *et al*. (2017)^(^ ^38^ ^)^ * Solomon Islands	Assessment of factors associated with obesity Case–control	Fifty-seven controls aged 20 years or above	24H-FR	AI, PC, BE		
Solomon Islands National Statistics Office (2015)^(^ ^28^ ^)^ Solomon Islands	HIES Cross-sectional	4479 households	Two-week food diary and expenditure	EX	EX	
Aswani and Furusawa (2007)^(^ ^39^ ^)^ Solomon Islands	Comparative study between marine protected areas and not Cross-sectional	Twenty households from six villages; participants aged 15 years or above	24H-FR	AI		
Konishi *et al*. (2011)^(^ ^40^ ^)^ Tonga	Assessment of nutrient intake and food sources Cross-sectional	Thirty-four adults aged 40–59 years	Two seven-consecutive-day 24H-FR in two different seasons; food weighing	AI, MS, PC		
Statistics Department (2010)^(^ ^29^ ^)^ Tonga	HIES Cross-sectional	1983 households	Two-week food diary and expenditure	EX	EX	
Central Statistics Division (2010)^(^ ^30^ ^)^ Tuvalu	HIES Cross-sectional	541 households	Two-week food diary and expenditure	EX	EX	
Vanuatu Ministry of Health 2017 (unpublished results) Vanuatu	Assessment of salt intake Cross-sectional	Adults aged 18–69 years; *n* 483	Spot urine sample and behaviours questionnaire			AI, BE
Martyn *et al*. (2015)^(^ ^43^ ^)^ Vanuatu	Identification of household factors associated with nutrition Modelling	Data from HIES: 3975 households from thirty islands	Data on household food expenditure from HIES	AI, MS	EX, PC	AI, MS
Vanuatu National Statistics Office (2012)^(^ ^31^ ^)^ Vanuatu	HIES Cross-sectional	3975 households	Two-week food diary and expenditure	EX	EX	EX
Estime *et al*. (2014)^(^ ^44^ ^)^ Multiple countries	Examination of links between trade and NCD Secondary analysis of data	Data from five countries: Samoa, *n* 2012; Solomon Islands, *n* 3822; Tonga, *n* 1640; Kiribati, *n* 1161; Vanuatu, *n* not reported (households)	Secondary analysis of household-level food expenditure and consumption data		EX, PC	
Micha *et al*. (2014)^(^ ^46^ ^)^ Multiple countries	Estimation of global, regional and national dietary fat intake Modelling	Data from 266 surveys in adults from 113 countries	Data from previous surveys: Bayesian hierarchical modelling	PC		
Powles *et al*. (2013)^(^ ^45^ ^)^ Multiple countries	Estimation of global, regional and national salt intake Modelling	Data from 142 surveys of 24 h urinary Na and 103 dietary Na from sixty-six countries	Data from previous surveys; Bayesian hierarchical modelling			AI

CNMI, Commonwealth of the Northern Mariana Islands; FSM, Federated States of Micronesia; PNG, Papua New Guinea; NCD, non-communicable disease; HIES, Household Income and Expenditure Survey; 24H-FR, 24 h food recall; 24H-UNa, 24 h urine collection; EX, household expenditure; BE, dietary behaviour; AI, absolute intake; PC, percentage contribution to energy intake; MS, main sources; AV, availability.

* Only estimates for the control group were included.

### Quality of studies

**Fig. 2:**
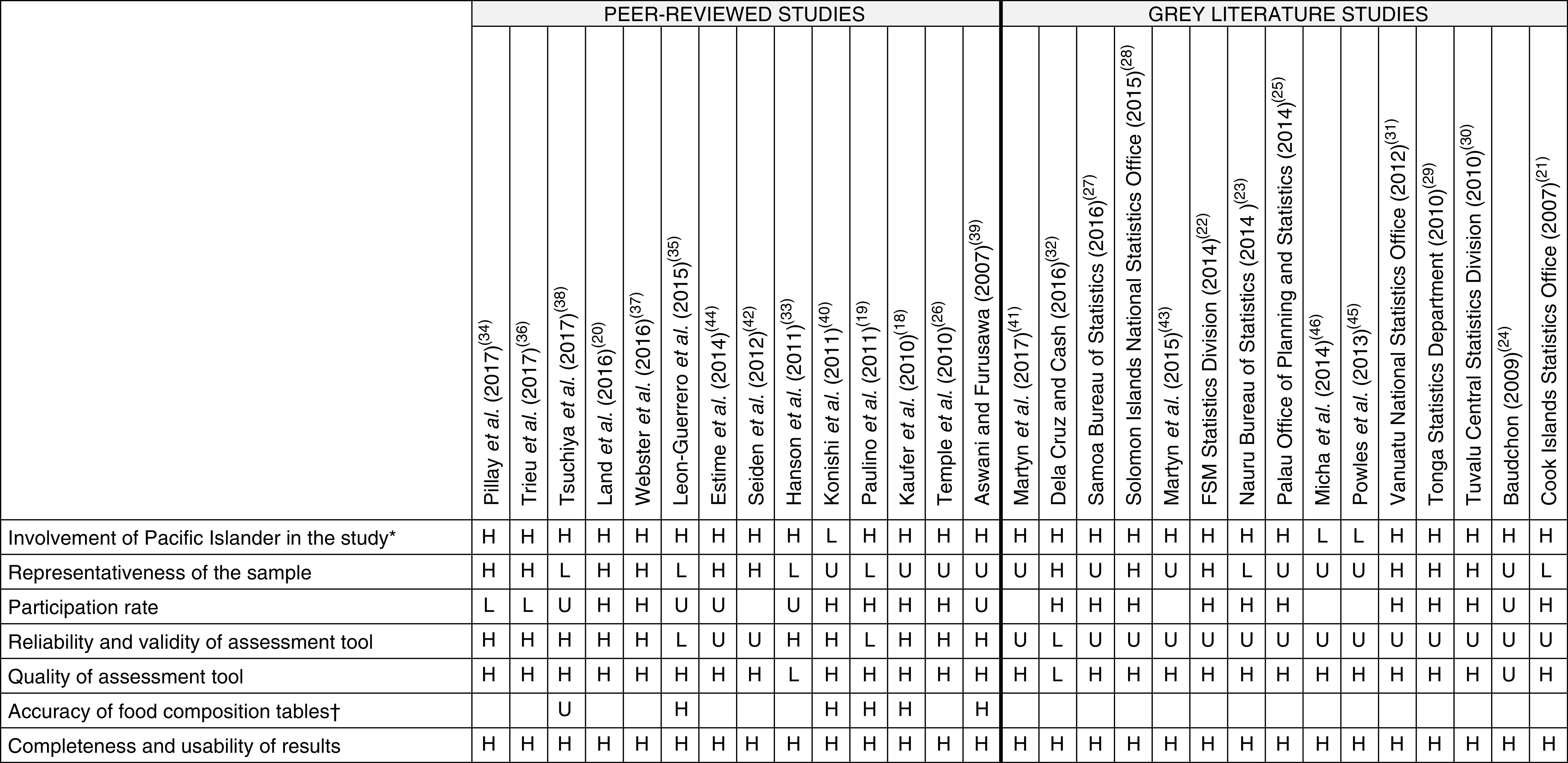
Summary of quality assessment (H, high quality; U, unclear quality; L, low quality; blank, not applicable) of studies included in the present systematic review on the contribution of fat, sugar and salt to diets in the Pacific Islands (note, quality assessment of two unpublished results was not completed: Fiji National Nutrition Survey 2017 and Vanuatu Ministry of Health 2017). *Refers to involvement during conception, design, conduct of research, including pre-testing and data collection, and distribution of results. †Assessed based on the appropriateness of the food composition database used to analyse the data, and whether a second person checked the linking of foods to the food composition table (FSM, Federated States of Micronesia)

A summary of the quality assessment of studies included in the current review is presented in Fig. 2. Only two studies^(^ ^20^ ^,^ ^37^ ^)^ were of high quality across all domains. The number of studies where quality was unclear or low was similar for peer-reviewed and grey literature in four domains: involvement of Pacific Islanders in the study (*n* 1 *v.* 2); representativeness of the sample (*n* 8 *v.* 9); quality of dietary assessment tool (*n* 1 *v.* 2); and completeness and usability of results (both *n* 0). The number of studies where quality was unclear or low based on participation rate was higher for peer-reviewed compared with grey literature (*n* 7 *v.* 1), while the opposite was true for reliability and validity of the dietary assessment tool (*n* 4 *v.* 15).

### Dietary assessment method and type of outcome data reported

Use of Household Income and Expenditure Survey (HIES) data, comprised of a two-week diary and records of expenditure on food, was the most common method for assessing dietary intake in studies (*n* 10), followed by 24 h food recall (*n* 6) and 24 h urine collection (*n* 4) and diet questionnaire (*n* 4). Other assessment methods employed were FFQ, dietary modelling, review of data from past surveys, food weighing, spot urine collection and food records. Five studies^(^ ^18^ ^,^ ^35^ ^,^ ^37^ ^,^ ^40^ ^)^ (one unpublished results: Vanuatu Ministry of Health 2017) used more than one method to measure dietary intake. Twenty-two studies published information on fat intake, seventeen on sugar intake and fourteen on salt intake. Overall, six types of outcomes regarding intake of fat, sugar or salt were reported: (i) absolute intake; (ii) household expenditure; (iii) percentage contribution to total energy intake; (iv) main sources; (v) availability (i.e. foods available for consumption, per capita); and (vi) dietary behaviours.

Absolute intake, main sources and percentage contribution of fat, sugar and salt to total energy intake were commonly measured by 24 h food recall, FFQ or dietary modelling. To measure salt intake, four studies from two countries (one in Fiji and three in Samoa)^(^ ^20^ ^,^ ^34^ ^,^ ^36^ ^,^ ^37^ ^)^ utilized the gold standard 24 h urine collection, while others employed spot urine collection and salt weighing. Household expenditure was measured using HIES data. Information on availability or access was either from secondary data or dietary modelling, while behaviours were assessed through 24 h food recall, FFQ or diet questionnaire.

### Fat, sugar and salt intake

#### Absolute intake

Results for each outcome measure are presented in Table 2 (fat intake), Table 3 (sugar intake) and Table 4 (salt intake). Information on absolute intake of fat and salt was available in six and ten countries, respectively, while only one country (Guam)^(^ ^19^ ^)^ had data on absolute intake of added sugar (47 g/d). Compared with total fat intake in Solomon Islands (25 g/d)^(^ ^38^ ^)^, intake in other countries was high (mean intake of 99 g/d in Guam^(^ ^35^ ^)^, 97 g/d in Fiji (Fiji National Nutrition Survey 2017, unpublished results), 75 g/d in Vanuatu^(^ ^43^ ^)^, 63 g/d in FSM^(^ ^18^ ^)^, 107 g/d for males and 88 g/d for females in Tonga^(^ ^40^ ^)^).

**Table 2:** Fat intake by type of outcome reported and country

Type of intake data	Countries with data	Description of intake
Absolute intake*	FSM	Kaufer, 2010 (24H-FR; adult women): 2005 (pre-intervention) 61·3 g/d; 2007 (post-intervention) 62·6 g/d
	Fiji	National Nutrition Survey, 2017 (24H-FR; adults): 97·1 (95 % CI 96·0, 98·2) g/d
	Guam	Leon-Guerrero, 2015 (FFQ; adults aged 18–61 years): total fat 98·9 (sd 67·7) g/d; saturated fat 33·0 (sd 24·0) g/d; MUFA 35·7 (sd 24·6) g/d; PUFA (sd 14·8) 21·4 g/d
	Solomon Islands	Tsuchiya, 2017 (24H-FR; controls aged ≥20 years): 25·4 (sd 13·2) g/d Aswani, 2007 (24H-FR; adults aged ≥15 years): males – Kozou 26·4 (sd 4·6) g/d; Nusa Hope 25·0 (sd 2·7) g/d; Baraulu 33·7 (sd 4·3) g/d; Olive 25·5 (sd 3·0) g/d; Dunde 41·6 (sd 3·8) g/d; Nusa Banga 19·7 (sd 4·0) g/d; females – Kozou 16·4 (sd 3·6) g/d; Nusa Hope 35·7 (sd 5·0) g/d; Baraulu 31·2 (sd 2·8) g/d; Olive 25·0 (sd 2·8) g/d; Dunde 42·8 (sd 6·8) g/d; Nusa Banga 25·6 (sd 14·5) g/d
	Tonga	Konishi, 2011 (24H-FR; adults aged 40–59 years; median intake): males 107·0 g/d; females 88·0 g/d
	Vanuatu	Martyn, 2015 (modelling): 75·0 g/d; rural 73·0 g/d; urban 81·0 g/d
Household expenditure	Cook Islands	2007: 3·4 %
	FSM	2014: 0·9 %
	Nauru	2014: 2·0 %
	New Caledonia	2009: 1·5 %
	Palau	2014: 2·0 %
	Samoa	2016: 0·9 %
	Solomon Islands	2015: 0·7 %
	Tonga	2010: 3·1 %
	Tuvalu	2010: 3·2 %
	Vanuatu	2012: 0·01 %
Main sources	Fiji	National Nutrition Survey, 2017 (24H-FR; adults): fish and seafood (fresh, canned, mixed food) 22·3 %, animal-source foods (poultry, mixed food, fresh meat and sausages) 18·8 %, wheat flour products (crackers, roti, instant noodles, sweets) 15·1 %, vegetable fat 8·6 %, coconut products 8·4 %, starchy staples 5·4 %
	Samoa	Martyn, 2017 (modelling): coconuts (popo) 19·0 %, chicken pieces 13·0 %, canned mackerel 8·0 %, cooking oil 6·0 % Seiden, 2012 (secondary analysis): coconuts and copra, vegetable oils, bovine meat, mutton and goat meat, pig meat, poultry meat
	Tonga	Konishi, 2011 (24H-FR; adults aged 40–59 years): imported foods contributed 65 % of total daily fat intake
	Vanuatu	Martyn, 2015 (modelling): coconut or copra, cooking oil, peanuts, cabin biscuits, fresh beef, chicken
Percentage contribution to energy†	FSM	Micha, 2014 (modelling; saturated fat): 22·9 % Kaufer, 2010 (24H-FR; adult women): 2005 (pre-intervention) 23·3 %; 2007 (post-intervention) 26·2 %
	Fiji	Micha, 2014 (modelling; saturated fat): 23·8 %
	Guam	Paulino, 2011 (24H-FR; women aged ≥40 years): total fat – feast day 34·1 (sd 7·8) %; non-feast day 27·5 (sd 9·6) %; saturated fat – feast day 11·4 (sd 4·7) %; non-feast day 7·9 (sd 3·4) %
	Kiribati	Micha, 2014 (modelling; saturated fat): 27·0 %
	PNG	Micha, 2014 (modelling; saturated fat): 23·2 %
	RMI	Micha, 2014 (modelling; saturated fat): 22·9 %
	Samoa	Micha, 2014 (modelling; saturated fat): 27·5 %
	Solomon Islands	Tsuchiya, 2017 (24H-FR; controls aged ≥20 years): 14·5 (sd 6·7) % Micha, 2014 (modelling; saturated fat): 23·9 %
	Tonga	Micha, 2014 (modelling; saturated fat): 22·8 % Konishi, 2011 (24H-FR; adults 40–59 years): males 33·0 (sd 4·0) %; females 31·0 (sd 3·0) %
	Vanuatu	Micha, 2014 (modelling; saturated fat): 25·7 %
Availability	Samoa	Martyn, 2017 (modelling): overall 96 g/d; Apia 101 g/d; Northwest Upolu 87 g/d; Rest of Upolu 99 g/d; Savaii 106 g/d Seiden, 2012 (secondary analysis): total fat availability increased by 73 % from 81 to 139 g/d
Behaviours	FSM	Hanson, 2011 (FFQ; adults): frequency of consumption of turkey tails 0·8 d/week; fried foods 2·5 d/week; percentage consuming fried foods (often to daily) 38·0 % Kaufer, 2010 (FFQ; adult women): frequency of consumption of imported animal fat in 2005 2·1 d/week; in 2007 2·4 d/week
	Solomon Islands	Tsuchiya, 2017 (24H-FR; controls aged ≥20 years): 82·5 % consumed oils and fats daily

FSM, Federated States of Micronesia; PNG, Papua New Guinea; RMI, Republic of the Marshall Islands; 24H-FR, 24 h food recall.

* Values reported as mean total fat intake, unless stated otherwise;

† Values reported as percentage contribution of total fat to energy, unless stated otherwise.

**Table 3:** Sugar intake by type of outcome reported and by country

Type of intake data	Countries with data	Description of intake
Absolute intake	Guam	Paulino, 2011 (24H-FR; women aged ≥40 years; added sugar): feast day 89·5 (sd 62·0) g/d; non-feast day 47·3 (sd 42·2) g/d
Household expenditure	Cook Islands	2007: 3·0 %†
	FSM	2014: 2·1 %‡
	Kiribati	2014: 13·0 %§
	Nauru	2014: 5·5 %║
	New Caledonia	2009: 4·7 %‡; 0·6 %§
	Palau	2014: 4·0 %‡
	Samoa	2016: 2·2 %‡
	Solomon Islands	2015, 2014: 2·6 %‡; 2·0 %§
	Tonga	2010: 4·2 %║
	Tuvalu	2010: 8·0 %‡
	Vanuatu	2015, 2012: 1·9 %§; 0·04 %║
Percentage contribution to energy*	Kiribati	Estime, 2014 (secondary analysis): 34·0 %
	Samoa	Martyn, 2017 (modelling; brown sugar): 8·0 %
	Solomon Islands	Estime, 2014 (secondary analysis): 2·0 %
	Vanuatu	Martyn, 2015 (modelling): 3·6 % Estime, 2014 (secondary analysis): 4·0 %
Behaviours	CNMI	Dela Cruz, 2016 (questionnaire; adults aged ≥18 years): about 75 % of adults drink one or more SSB daily
	FSM	Hanson, 2011 (FFQ; adults): percentage consuming imported sugar foods (often to daily) 16·0 %; imported drinks with sugar (often to daily) 72·0 % Kaufer, 2010 (FFQ; adult women): frequency of consumption of sugar (imported product or added to local food) in 2005, 3·2 d/week; in 2007, 1·9 d/week

FSM, Federated States of Micronesia; CNMI, Commonwealth of the Northern Mariana Islands; 24H-FR, 24 h food recall; SSB, sugar-sweetened beverage.

* Values reported as percentage contribution of sugar to energy, unless stated otherwise.

† Confectionery.

‡ Sugar, jam, honey, chocolate, confectionery.

§ Sugar alone.

║ Sugar and confectionery.

**Table 4:** Salt intake by type of outcome reported and by country

Type of intake data	Countries with data	Description of intake
Absolute intake*	FSM	Powles, 2013 (modelling): overall 6·5 g/d; males 6·8 g/d; females 6·2 g/d
	Fiji	National Nutrition Survey, 2017 (24H-FR; adults): 8·9 g/d Pillay, 2017 (24H-UNa; adults aged 25–64 years): baseline 11·7 (se 0·7) g/d; follow-up 10·3 (se 0·5) g/d Powles, 2013 (modelling): overall 7·3 g/d; males 7·7 g/d; females 7·0 g/d
	Guam	Leon-Guerrero, 2015 (FFQ; adults aged 18–61 years): 10·1 (sd 6·6) g/d
	Kiribati	Powles, 2013 (modelling): overall 5·6 g/d; males 5·9 g/d; females 5·4 g/d
	PNG	Powles, 2013 (modelling): overall 6·2 g/d; males 6·6 g/d; females 5·9 g/d Temple, 2009 (salt weighing; mean per capita salt consumption): 5·6 (sd 1·5) g/d
	RMI	Powles, 2013 (modelling): overall 6·5 g/d; males 6·8 g/d; females 6·2 g/d
	Samoa	Trieu, 2017 (24H-UNa; adults aged 18–64 years): baseline 7·3 (se 0·3) g/d; follow-up 7·5 (se 0·2) g/d Webster, 2016 (24H-UNa; adults aged 18–64 years): overall 7·1 (se 0·2) g/d; males 7·6 (se 0·3) g/d; females 6·4 (se 0·1) g/d Land, 2016 (24H-UNa; women aged 18–45 years): 6·6 (sd 3·2) g/d Powles, 2013 (modelling): overall 5·3 g/d; males 5·5 g/d; females 5·0 g/d
	Solomon Islands	Powles, 2013 (modelling): overall 5·9 g/d; males 6·2 g/d; females 5·6 g/d
	Tonga	Powles, 2013 (modelling): overall 6·9 g/d; males 7·3 g/d; females 6·6 g/d
	Vanuatu	Ministry of Health, 2017 (spot urine; adults aged 18–64 years): overall 7·2 (sd 2·3) g/d; males 7·8 (sd 2·6) g/d; females 6·5 (sd 1·7) g/d Martyn, 2015 (modelling): overall 5·3 g/d; rural 4·9 g/d; urban 6·6 g/d Powles, 2013 (modelling): overall 5·7 g/d; males 5·9 g/d; females 5·4 g/d
Household expenditure	Cook Islands	2007: 0·1 %
	Vanuatu	2012: 1·8 %
Main sources	Fiji	National Nutrition Survey, 2017 (24H-FR; adults): wheat flour products (roti, breakfast crackers, bread, homemade flour products) 27·6 %, fish and seafood 16·8 %, animal-source foods 16·2 %, salt 13·7 %, savoury snacks 8·2 %
	Samoa	Martyn, 2017 (modelling): table salt 39·0 %, instant noodles 9·0 %, canned mackerel 9·0 %
	Vanuatu	Martyn, 2015 (modelling): bread contributed 15·4 % of Na available, water taro 7·5 %, cabin biscuits 7·4 %, tinned tuna 7·0 %
Availability	Samoa	Martyn, 2017 (modelling): overall 7·4 g/d; Apia 7·8 g/d; Northwest Upolu 7·3 g/d; Rest of Upolu 7·6 g/d; Savaii 7·0 g/d
Behaviours	CNMI	Dela Cruz, 2016 (questionnaire; adults aged ≥18 years): two-thirds consume at least one serving of processed meats daily
	FSM	Hanson, 2011 (FFQ; adults): frequency of consumption of imported salty foods 0·7 d/week; percentage consuming salty foods (often to daily) 8·0 %
	Samoa	Webster, 2016 (questionnaire; adults aged 18–64 years): avoid processed food 58·7 %, look at Na labels on food 42·6 %, do not add salt on the table 43·8 %, buy low-salt alternatives 54·9 %, do not add salt when cooking 49·0 %, use spices other than salt 43·9 %, avoid eating out 60·9 %
	Vanuatu	Ministry of Health, 2017 (questionnaire; adults aged18–64 years): avoid processed food 76·6 %, look at Na labels on food 32·1 %, do not add salt on the table 60·7 %, buy low-salt alternatives 69·4 %, do not add salt when cooking 74·2 %, use spices other than salt 61·1 %, avoid eating out 16·2 %

FSM, Federated States of Micronesia; PNG, Papua New Guinea; RMI, Republic of the Marshall Islands; CNMI, Commonwealth of the Northern Mariana Islands; 24H-FR, 24 h food recall; 24H-UNa, 24 h urine collection.

* Values were converted to salt in g/d (393·4 mg sodium=1 g salt).

Of the ten countries with data on absolute salt intake, only two (Fiji and Samoa) were based upon 24 h urine collection. Four countries (Fiji, PNG, Samoa and Vanuatu) had multiple data points, but differences in study design, assessment methods and sampling did not allow for assessment of change over time. Five countries (FSM, Kiribati, RMI, Solomon Islands and Tonga) had salt intake estimates solely based from modelling^(^ ^45^ ^)^. The most recent data on intake of fat, sugar and salt, and the dietary assessment method used, are shown in Table 5.

**Table 5:** Most recent fat, sugar and salt intake estimates and dietary assessment method used

Country	Year	Intake (g/d)		Dietary assessment method
Fat intake				
FSM	2005	62·6		24H-FR
Fiji	2017	97·1	95 % CI 96·0, 98·2	24H-FR
Guam	2015	98·9	sd 67·7	FFQ
Solomon Islands	2017	25·4	sd 13·2	24H-FR
Tonga	2011	107·0 M 88·0 F	sd 21·0 sd 20·0	24H-FR
Vanuatu	2015	75·0		Dietary modelling
Sugar intake				
Guam	2011	47·3	sd 42·2	24H-FR
Salt intake				
FSM	2013	6·5	95 % CI 5·5, 7·7	Dietary modelling
Fiji	2017	10·3	se 0·5	24H-UNa
Guam	2015	10·1	sd 6·6	FFQ
Kiribati	2013	5·6	95 % CI 4·6, 6·8	Dietary modelling
Papua New Guinea	2013	6·2	95 % CI 5·3, 7·3	Dietary modelling
RMI	2013	6·5	95 % CI 5·5, 7·7	Dietary modelling
Samoa	2017	7·5	se 0·2	24H-UNa
Solomon Islands	2013	5·9	95 % CI 5·0, 7·0	Dietary modelling
Tonga	2013	6·9	95 % CI 5·8, 8·1	Dietary modelling
Vanuatu	2017	7·2	sd 2·3	Spot urine collection

FSM, Federated States of Micronesia; RMI, Republic of the Marshall Islands; M, males; F, females; 24H-FR, 24 hour food recall; 24H-UNa, 24 h urine collection.

#### Household expenditure

Ten countries had household expenditure data on fats and oils, which ranged from 0·01 % in Vanuatu^(^ ^31^ ^)^ to 3·4 % in Cook Islands^(^ ^21^ ^)^. On the other hand, eleven countries had expenditure data on sugar although how it was reported varied, with studies reporting on sugar alone (*n* 4), sugar and confectionery (*n* 3), sugar, jam, honey, chocolate and confectionery (*n* 6), and one on confectionery alone. Expenditure on sugar, jam, honey, chocolate and confectionery ranged from 2·1 % in FSM to 8·0 % in Tuvalu. Expenditure on sugar alone ranged from 0·6 % in New Caledonia^(^ ^24^ ^)^ to 13·0 % in Kiribati^(^ ^44^ ^)^. Only two countries (Cook Islands and Vanuatu) had data on household expenditure on salt (0·1^(^ ^21^ ^)^ and 1·8 %^(^ ^31^ ^)^, respectively).

#### Sources of fat, sugar and salt

Four countries had data on sources of fat and these were coconut products, oils, meat products and fish products. A study in Tonga^(^ ^40^ ^)^ reported that imported food products including meats and processed foods contributed 65 % of total daily fat intake. Three countries had data on sources of salt. Bread (15·4 %) was found to be the highest contributor of dietary salt in Vanuatu^(^ ^43^ ^)^, while table salt was common in Fiji (13·7 %; Fiji National Nutrition Survey 2017, unpublished results) and Samoa (39·0 %)^(^ ^41^ ^)^. Meat and fish products were consistently among the top sources of salt in these three countries. There were no data on the sources of sugar, but studies in CNMI and FSM reported frequent consumption of sugar-sweetened beverages^(^ ^32^ ^,^ ^33^ ^)^.

#### Percentage contribution to energy intake

Percentage contribution of total fat to total energy intake varied across the four countries with data (14·5 % in Solomon Islands^(^ ^38^ ^)^, 23·3 % in FSM^(^ ^18^ ^)^, 27·5 % in Guam^(^ ^19^ ^)^, 33 % for males and 31 % for females in Tonga^(^ ^40^ ^)^). Nine countries estimated percentage contribution of saturated fat to total energy intake from modelling and it ranged from 22·8 % in Tonga to 27·5 % in Samoa^(^ ^46^ ^)^. A study in Guam^(^ ^19^ ^)^ reported the percentage contribution of saturated fat on a regular day (7·9 %) and a feast day (11·4 %) based on 24 h food recall. On the other hand, percentage contribution of sugar to total energy intake appeared similar in two countries (3·6 % in Vanuatu^(^ ^43^ ^)^ and 2 % in Solomon Islands^(^ ^44^ ^)^) but was notably high (34 %) in Kiribati^(^ ^44^ ^)^. A study in Samoa reported that brown sugar contributed 8 % to total energy intake but did not provide information on total sugar^(^ ^41^ ^)^.

#### Availability of fat, sugar and salt for consumption (per capita)

There was very little information on availability of fat, sugar and salt. In fact, only Samoa had data on this outcome which showed that there was high availability of fats in the country as well as excessive access to sodium^(^ ^41^ ^,^ ^42^ ^)^.

#### Dietary behaviours

Dietary behaviour data from FSM and Solomon Islands indicated that foods high in fats and oils were consumed frequently^(^ ^18^ ^,^ ^33^ ^,^ ^38^ ^)^. Sugar was also consumed frequently with 75 %^(^ ^32^ ^)^ and 50 %^(^ ^33^ ^)^ of adults in CNMI and FSM, respectively, reporting drinking one or more sugar-sweetened beverages per day. Studies in Samoa^(^ ^37^ ^)^ and Vanuatu (Vanuatu Ministry of Health 2017, unpublished results) using the WHO STEPwise approach to surveillance of NCD risk factors (STEPS) instrument^(^ ^47^ ^)^ reported salt-related behaviours including not adding salt at the table (44 and 61 %), not adding salt when cooking (49 and 74 %), avoiding processed foods (59 and 77 %), looking at Na labels on foods (43 and 32 %), buying low-salt alternatives (55 and 69 %), using spices other than salt (44 and 61 %) and avoiding eating out (61 and 16 %).

## Discussion

The current systematic review found that while dietary data were available on fat, sugar and salt intakes in Pacific Island countries, some countries had limited or no data, and most countries lacked high-quality data. In fact, only Fiji has a recent comprehensive nutrition survey. The absence of quality nutrition data is concerning, given high rates of NCD in the region^(^ ^1^ ^)^ and changing dietary patterns from traditional diets toward a more Western-style diet as shown by historical trends from previous studies^(^ ^4^ ^,^ ^6^ ^)^. Furthermore, changes in local food production, population growth, environmental conditions, increased reliance on processed and imported foods, and lack of policies^(^ ^12^ ^,^ ^48^ ^)^ also put Pacific Islanders at a higher risk for NCD. There is a need for better data on Pacific diets to establish benchmark consumption levels, to better inform initiatives to improve diets. Also, quality parameters should be considered when interpreting existing data.

The percentage contribution to total energy intake from fat was below the recommended 30–35 % of total energy^(^ ^49^ ^)^ for four countries, although in the case of Solomon Islands^(^ ^38^ ^)^ energy from fat was at the minimum level of 15 % recommended for adequate population consumption of energy^(^ ^49^ ^)^. Furthermore, it is important to note that absolute intake of total fat was about 100 g/d in Fiji, Guam and Tonga, similar to intakes reported in developed countries^(^ ^50^ ^)^. In terms of saturated fat intake, the modelling study by Micha *et al*.^(^ ^46^ ^)^ showed that the nine Pacific Island countries with data had the highest intakes of this nutrient (average of 23·5 %), far exceeding the recommended limit of <10 % of total energy^(^ ^49^ ^)^ and the global average of 9·4 %. A review of data from forty countries in 2013 found that total fat intake is correlated with saturated fat intake, but not with polyunsaturated fat intake^(^ ^51^ ^)^. This suggests that interventions aimed at lowering fat intake should focus not only on lowering saturated fat intake, but also increasing polyunsaturated fat intake^(^ ^51^ ^,^ ^52^ ^)^.

There was not enough information about absolute intake of sugar, although based on household expenditure and percentage contribution to energy intake data it appeared that Kiribati had high sugar intakes relative to other Pacific Island countries. Unfortunately, there was no information available about sources of sugar or consumption patterns to further explain this finding, although an earlier study reported that sugar-sweetened beverage consumption in Kiribati was moderate (less than 23 % of 11- to 18-year-olds consuming soft drinks daily and import of less than 40 litres per person^(^ ^8^ ^)^).

All countries with data appeared to be exceeding the WHO recommended limit of less than 5 g salt/person per day, contributing to the high rates of morbidity and mortality due to CVD^(^ ^53^ ^)^. As such, salt reduction initiatives and policies are essential to lower population salt intake and reduce the burden of NCD^(^ ^54^ ^)^. A systematic review of salt reduction initiatives around the world in 2015 reported that only a few Pacific Island countries engage in actions to reduce salt intake (e.g. food reformulation and front-of-pack labelling)^(^ ^55^ ^)^, and a separate review showed that while there has been good progress in terms of planning strategies, barriers such as the lack of capacity to accurately measure salt intake and effectively implement activities hinder further progress^(^ ^56^ ^)^. The population-based multisectoral salt reduction interventions implemented in Fiji^(^ ^34^ ^)^ and Samoa^(^ ^36^ ^)^ both showed a non-significant change in salt intake following the interventions. The authors identified reasons for the null effect such as the short intervention duration (18 months), low intervention dose, lack of participation from other government sectors and limited engagement with the food industry. These suggest the need for stronger and more active mechanisms for multisectoral governance and partnership; engaging with the food industry; and planning, monitoring and evaluation of interventions to reduce salt intake in the region.

Although not specifically covered in the present review, a common theme noted in the identified studies was the increasing reliance of Pacific Islanders on processed foods. This was evident in countries with data on main sources and behaviours, which showed that the consumption of these foods (e.g. canned meat and fish, rice, sugar-sweetened drinks and instant noodles, among others) was frequent and common. This trend may also be reflected at feasts. Paulino *et al*.^(^ ^19^ ^)^ found that the type of foods served during feast days in Guam has shifted from low-energy foods to predominantly high-energy foods such as ‘meats, desserts, sodas and other sweetened beverages’. Further studies are warranted to confirm this in other countries. A review of processed foods in Pacific Island countries reported that most are heavily dependent upon food imports with only a small proportion of foods produced locally^(^ ^48^ ^)^. Previous studies have shown that an increasing reliance on energy-dense nutrient-poor imported foods is associated with increases in NCD^(^ ^15^ ^,^ ^57^ ^,^ ^58^ ^)^. This demonstrates the wide scope for potential strategies including regulations on imported products, food safety and labelling in the Pacific Islands^(^ ^48^ ^,^ ^59^ ^)^.

The current review revealed a range of different dietary assessment methods employed by studies. This variation limits comparison of intakes between countries and over time within a country. Data were available from HIES however, including in five countries where HIES was the only source of nutrition data. In the absence of other national surveys using standardized methodologies, these surveys may provide a good proxy measure of dietary intake^(^ ^60^ ^)^. If HIES are used in this manner, standardization and reporting of methods may need to be strengthened as most reports did not provide information on standardization methods. We also observed country differences in the way foods were grouped and reported which made them unsuitable for comparison. For instance, sugar intake was reported as sugar alone or confectionery alone, sugar and confectionery combined, or sugar, jam, honey, chocolate and confectionery.

Differences in quality between peer-reviewed studies and grey literature studies were observed. Peer-reviewed studies generally used direct approaches to measuring diet but had poor participation rates, while grey literature studies, which were generally reports of national surveys, had better participation rates but often employed indirect approaches to measure dietary intake. Furthermore, grey literature studies were larger scale (national) compared with peer-reviewed studies which were usually implemented at a community or village level. This suggests that strengthening the quality of nutrition data in the Pacific will require the use of more direct dietary methods in national surveys and efforts to link large- and small-scale studies. This was achieved in Samoa^(^ ^37^ ^)^ where 24 h urine collection was measured from a subset of STEPS participants (i.e. targeted 500 individuals out of 2800 STEPS participants) and demonstrated that it was feasible to incorporate salt intake measurements into a population-based national survey without large additional costs. This is important since the WHO has recommended the integration of salt intake monitoring through urine collection into its STEPS surveys^(^ ^47^ ^)^.

Strengths of the present review include the systematic approach, the breadth of databases searched, multiple search strategies and extensive grey literature searches, and stakeholder input. All Pacific Island countries were individually searched within the published literature. Quality assessment was conducted at the dietary assessment and study methodology level, which, to the authors’ knowledge, has not been done before in a review of dietary intakes of Pacific Island countries. Limitations of the review include the restriction by language and publication date. Given several of the countries included are French-speaking and that all Pacific Island countries have English as a second language, it is likely that nutrition data are reported in other languages that we have missed. Synthesis was also only based on studies that collected data in 2005 and thereafter, and a meta-analysis would have strengthened the analysis but was deemed inappropriate due to the heterogeneity across the studies. Within each type of outcome, it was also not possible to pool the results due to different assessment methods and analytical approaches. The review also focused on adults only. A number of Pacific countries are implementing health promotion programmes in schools, so a similar review of children and adolescents may be helpful.

## Conclusions

In summary, the present study reviews the availability and quality of information on dietary fat, sugar and salt intake in the Pacific Islands. Although varying methods and quality meant that it was difficult to assess and compare studies, the review suggests that intakes of fat, sugar and salt are generally high in countries across the region, with the exception of relatively low salt and fat intakes measured in Solomon Islands. More research of high quality is required throughout the Pacific Islands in order to understand health risks attributable to the diets of its people and inform effective policy interventions.

## Supplementary material

For supplementary material accompanying this paper visit https://doi.org/10.1017/S1368980018003609. click here to view supplementary material
