# Supplementary material for: Contribution of fat, sugar and salt to diets in the Pacific Islands: a systematic review
Source: Public Health Nutr. 2019 Jan 7;22(10):1858–71. doi: 10.1017/S1368980018003609 (PMC6670018; doi:10.1017/S1368980018003609)
Supplement: Supplementary file 1 [file S1368980018003609sup001.docx]

**Supplemental Table 1.** Quality assessment tool for dietary intake studies

| **Quality Domain** | **Ratings and explanations** | |
| --- | --- | --- |
| **Involvement of Pacific Islander in the study** | **Low quality** | Limited involvement from Pacific Islander people and/or community representatives during conception, design, conduct of research; including pre-testing and data collection and distribution of results. |
|  | **High quality** | Pacific Islander people and/or community representatives involved in the study during conception, design, conduct of research; including pre-testing and data collection and distribution of results. |
|  | **Unclear** | Insufficient detail reported to determine input from Pacific Islander people and/or communities during conception, design and conduct of research; including pre-testing and data collection. |
| **Representativeness of sample underlying population** | **Low quality** | Study sample differs from target/underlying population, due to biased patterns of response and/or participation: e.g. convenience sample or volunteers. |
|  | **High quality** | Study sample resembles underlying population (target population). This could incorporate the application of sampling weights, and/or comparing the sample to a census (or similar) to show similarity with target population. |
|  | **Unclear** | Insufficient detail reported to determine how selection took place and representativeness of target population. |
| **Participation rate**  **50% at each study site where applicable* | **Low quality** | Less than 50%* and no attempt to quantify characteristics of non-responders. |
|  | **High quality** | Greater than 50%* or attempt to quantify characteristics of non-responders with appropriate adjustments made to the results. |
|  | **Unclear** | Insufficient information reported to determine participation rate. |
| **Reliability and validity of dietary assessment tool** | **Low quality** | Dietary assessment measure that has not been validated in the sample population; without convincing text regarding the validation within the sample population. |
|  | **High quality** | Dietary assessment measure validated in sample population/with convincing text regarding validation in the sample population. |
|  | **Unclear** | Not enough information provided on the validity of the dietary assessment measure. |
| **Quality of dietary assessment tool**    *See criteria below* | **Low quality** | The dietary assessment measure scores negatively according to the criteria for quality of the dietary assessment method. |
|  | **High quality** | The dietary assessment measure scores positively according to the criteria for quality of the dietary assessment method. |
|  | **Unclear** | Insufficient information reported to determine the quality of the dietary assessment measure. |
| **Accuracy of food composition tables** | **Low quality** | An inappropriate food composition database has been used for the analysis of food consumption data and was not checked by a second person. |
|  | **High quality** | An appropriate food composition database was used for the analysis of food composition data and a second person checked the linking of foods to the food composition table. |
|  | **Unclear** | Insufficient information reported to determine data analysis methods. |
| **Completeness and usability of results** | **Low quality** | Not all pre-specified primary outcomes have been reported. Outcome of interest reported but incomplete or unclear/not usable format: e.g. mean but no error estimate (SD or 95% CI). |
|  | **High quality** | All pre-specified outcomes of interest reported in a complete/useable format. |
|  | **Unclear** | Insufficient information provided. |

***Criteria for quality of the dietary assessment tool:***

Overall rating ≤50% corresponds to a low quality, >50% is a high quality; subtract the number of NA categories from the denominator before calculating the percentage.

1. Is the method appropriate for the question being asked? (for sodium: complete 24-hour urine collection; validated dietary survey and spot urine could also score low risk of bias with convincing text regarding reliability and validity)
2. Is the description of the method sufficient to judge whether the method is likely to be used correctly?
3. Does the assessment cover an appropriate time frame based on the dietary assessment method used?
4. For studies where nutrient intakes are presented, have foods been translated to nutrient intakes appropriately (e.g. enough information on portion sizes)?
5. Has seasonality been taken into account appropriately?
6. Have appropriate visual aids or prompts been used?
7. Does the analysis account for potential confounding factors?

This quality assessment tool was adapted from a systematic review of diets of Aboriginal and Torres Strait Islander peoples in Australia.

**Source:** Whalan S, Farnbach S, Volk L, et al. What do we know about the diets of Aboriginal and Torres Strait Islander peoples in Australia? A systematic literature review. *Australian and New Zealand Journal of Public Health.* 2017;41(6):579-584.
